# Carrier Multiplication Mechanisms and Competing Processes in Colloidal Semiconductor Nanostructures

**DOI:** 10.3390/ma10091095

**Published:** 2017-09-18

**Authors:** Stephen V. Kershaw, Andrey L. Rogach

**Affiliations:** Department of Materials Science and Engineering and Centre for Functional Photonics (CFP), City University of Hong Kong, Hong Kong S.A.R., China; andrey.rogach@cityu.edu.hk

**Keywords:** quantum dots, carrier multiplication, hot carrier cooling processes, carrier dynamics

## Abstract

Quantum confined semiconductor nanoparticles, such as colloidal quantum dots, nanorods and nanoplatelets have broad extended absorption spectra at energies above their bandgaps. This means that they can absorb light at high photon energies leading to the formation of hot excitons with finite excited state lifetimes. During their existence, the hot electron and hole that comprise the exciton may start to cool as they relax to the band edge by phonon mediated or Auger cooling processes or a combination of these. Alongside these cooling processes, there is the possibility that the hot exciton may split into two or more lower energy excitons in what is termed carrier multiplication (CM). The fission of the hot exciton to form lower energy multiexcitons is in direct competition with the cooling processes, with the timescales for multiplication and cooling often overlapping strongly in many materials. Once CM has been achieved, the next challenge is to preserve the multiexcitons long enough to make use of the bonus carriers in the face of another competing process, non-radiative Auger recombination. However, it has been found that Auger recombination and the several possible cooling processes can be manipulated and usefully suppressed or retarded by engineering the nanoparticle shape, size or composition and by the use of heterostructures, along with different choices of surface treatments. This review surveys some of the work that has led to an understanding of the rich carrier dynamics in semiconductor nanoparticles, and that has started to guide materials researchers to nanostructures that can tilt the balance in favour of efficient CM with sustained multiexciton lifetimes.

## 1. Introduction

**Figure 1:**
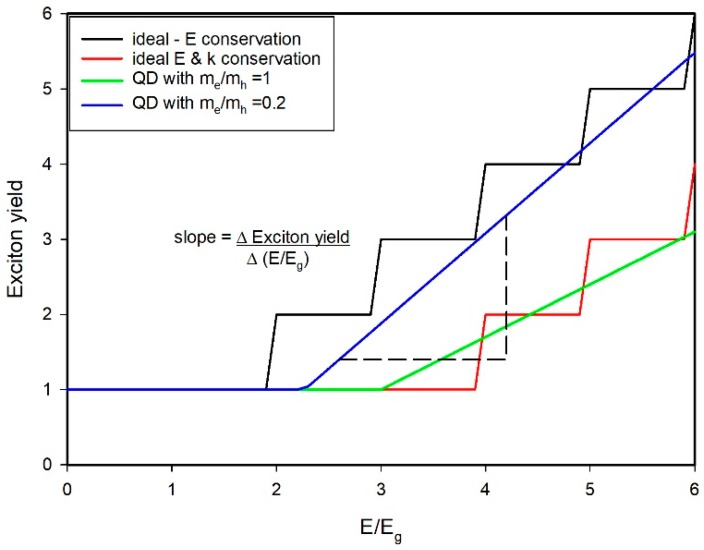
A staircase-like increase is shown in the ideal case for carrier multiplication (CM) where only energy conservation applies (black curve) and where momentum conservation must also be satisfied (red curve). In the latter case, the threshold corresponds to that of a semiconductor similar to bulk silicon (=4 E_g_). In practice, quantum dots (QDs) do not show abrupt increases in exciton yields vs. excitation energy as shown for the green and blue curves. Their threshold may sometimes be termed ‘soft’. The green line would correspond to a QD with equal hole and electron effective masses (m_e_, m_h_ respectively), whilst the blue line shows a case where m_e_/m_h_ = 0.2.

The process of carrier multiplication (CM) in semiconductors follows excitation with a high energy photon where the excess of energy above the bandgap, E_g_, is at least twice the latter and may in fact need to be considerably higher for the multiplication process to occur. CM is sometimes also termed multiple exciton generation (MEG) where the excitation process leads to the formation of multi-exciton states. The process is known to occur in both bulk [1,2] and quantum confined semiconductors, i.e., quantum dots (QDs), and the latter are understood to offer advantages over their bulk counterparts owing to potentially lower multiplication thresholds and higher slope efficiencies [3] (the rate at which the carrier or exciton yield increases with excitation photon energy once the threshold has been exceeded, see Figure 1). Excitation at high photon energies is said to initially yield hot carriers (excitons, electrons or holes) where the excess energy is partitioned between the carriers according to the ratio of the inverse of their effective masses as an optical selection rule [3]. The precise mechanism by which the excess energy is converted to multi-excitons has been the subject of frequent debate and we will discuss the various scenarios in more detail later. In bulk semiconductors, the kinetic impact ionization process is most frequently encountered and is often used as the model for CM in QDs by extrapolation from the bulk case, with modification where appropriate for momentum conservation considerations.

Impact ionization is well known in many bulk semiconductors and is the basis for established photodetector technology in avalanche photodiodes (APD): Si, Ge, InAs, InP, InGaAs, Ga_1−*x*_Al*_x_*Sb and Hg_(1−*x*)_Cd*_x_*Te have all been exploited for APD devices [4,5,6]. In the Ga_1−*x*_Al*_x_*Sb and Hg_(1−*x*)_Cd*_x_*Te alloys, advantage is taken of the ability to manipulate the spin-orbit splitting energy in order to place the split-off band at resonance with the energy of the bandgap to enhance the efficiency of the multiplication process for certain alloy compositions [4,6]. CM has also been envisaged as offering the scope for substantial improvements in solar cell performance. Unfortunately, in the case of many bulk materials, the threshold for multiplication is rather high. For an optimum bandgap energy for solar conversion (around 1.2–1.4 eV) [7], the CM threshold for bulk silicon of around 4 E_g_ [8,9,10] puts most of the solar spectrum beyond the range of the CM effect.

Beard et al. [11] have compared the threshold and slope efficiencies for bulk and QD semiconductors. They commented that for bulk PbSe and PbS the CM threshold should be 4 E_g_ (experimentally 4.5 E_g_) whereas the more recent experimental values for QD versions of these materials range between 2.7 E_g_ and 3 E_g_. However, they also commented that for significant benefit in solar cell performance, the threshold energy should be as low as 2 E_g_–2.5 E_g_ i.e., at or approaching the energy conservation limit. For PbSe QDs as an example, Beard et al. predicted that the Shockley-Queisser solar cell efficiency limit [12] could be raised to around 42% [11], though more recent evaluations have suggested that rather lower efficiencies might be available (e.g., Binks with experimental CM data shows very marginal solar cell efficiency improvements in the 1.2 eV–1.4 eV bandgap range [13], and Nair and et al. predicted realistic enhancements in power conversion efficiencies of under 5% [14] also based on reported CM performance at that time. The actual improvement in the performance of solar cell test devices has been somewhat limited. Semonin et al. [15] attributed only 4% of the photocurrent in their PbSe photovoltaic devices to CM. The rather muted outcomes from trying to use CM to enhance solar cell efficiencies have led to a continued interest in the alternate approach of trying to extract hot carriers before either cooling or fission (i.e., CM) of the hot exciton can occur [16,17]. Nonetheless, if CM with near energy conservation limit threshold and high slope efficiency can be obtained, then it remains a potentially interesting mechanism for improved solar cells along with other applications including QD based lasers, high brightness light emitting devices, etc. However, to be of practical benefit most of the carriers would have to be extracted within the respective multi-exciton lifetimes. This is due to the competing process of Auger non-radiative recombination which rapidly and sequentially annihilates excess excitons until the QDs are only single exciton occupied, or worse still leave the QDs in a charged state so that even subsequent single exciton excitation will be at the mercy of fast trion Auger non-radiative recombination [18,19]. For spherical QDs the biexciton lifetimes are typically a few 10s to 100 ps, for core/shell heterostructures several 100s or even 1000s of ps [20], for nanorods similarly 1000 ps is achievable [21], whilst in more recently explored 2D nanoparticles (nanoplatelets) biexcitons can survive for as long as 14,000 ps [22]. For higher multi-exciton occupancies, the triexciton and n-excitons decay even more rapidly than the biexciton [23] and so fast carrier extraction is a must for solar cell applications in order to reap the benefits of CM.

## 2. CM Quantum Yield Measurements

**Figure 2:**
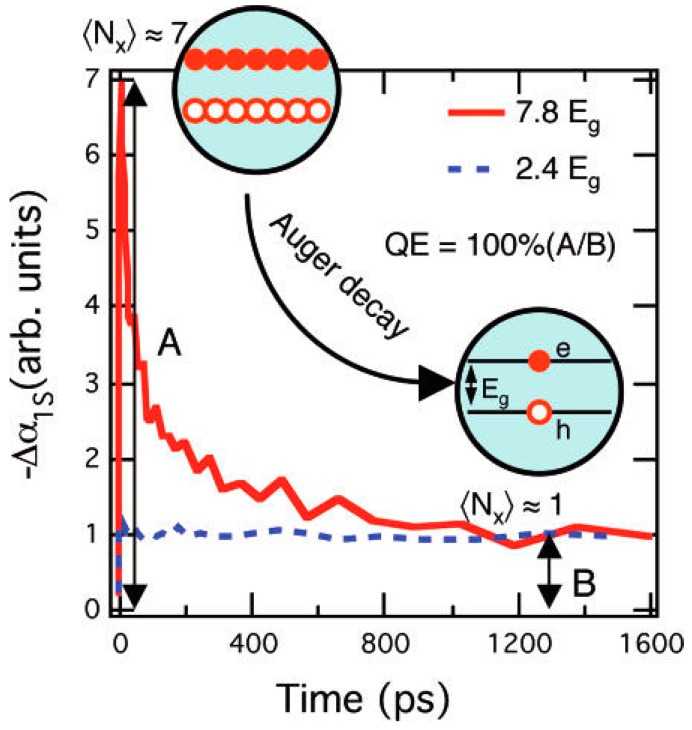
Calculations of CM quantum efficiency (QE) from time-resolved transient absorption (TA) data illustrated using two traces measured for PbSe nanocrystals (NCs) at low pump intensity (N_0_ < 0.1) with photon energies 2.4 E_g_ (no CM; dashed line) and 7.8 E_g_ (CM producing approximately seven excitons per NC; solid line). Reprinted with permission from ref. [28]. Copyright (2006) American Chemical Society.

Multi-excitons can exist in QDs either because a hot exciton has undergone fission to form two or more cooler excitons or simply because more than one separate photon absorption event has each yielded an exciton before the other(s) have decayed by radiative or non-radiative channels. The greater the excitation fluence the higher the probability that this may occur. Irrespective of how the multiexcitons were created in the nanoparticle, as soon as two or more excitons are in residence, Auger recombination may occur, progressively removing one exciton at a time until only a single exciton remains. In many cases, the latter has a much longer lifetime than any of the multi-excitons and this forms the basis for several measurement techniques that can follow an ensemble’s exciton occupancy. Femtosecond/picosecond transient absorption [24] (TA), heterodyne transient grating [25] (TG), picosecond transient photoluminescence [26] (TPL), and time resolved microwave conductivity [27] (TRMC) measurement techniques can all follow the evolution of the (average) exciton occupancy per QD. In the TA case for example, the band edge absorption is rapidly bleached as excitons populate the QD and cool towards the band edge. The intensity of the bleach is a measure (a linear function) of the number of excitons per QD. As Auger recombination progresses the bleach intensity falls to an almost asymptotic level, usually on the nanoseconds timescale, with the level corresponding to single exciton occupancy (e.g., Figure 2) [28]. On longer timescales, the bleach will recover completely as the much slower single exciton recombination channels return the QD to the neutral ground state. Similar dynamics are seen in TG signals. The peak number of excitons can therefore simply be determined by the ratio of the peak to single exciton asymptotic signal levels. To determine how many of the multiexcitons were formed by hot exciton fission (e.g., by impact ionization) rather than multiple absorption events, the experiment can be repeated several times for a range of different fluences, including very low levels and the zero fluence peak occupancy can be extracted from the fluence dependence.

Unfortunately, in practice things are not quite so simple. Whilst the above experimental method/analysis is correct in principle, it has been suggested that there is a possible additional contribution to the picosecond decay that may masquerade as Auger multiexciton decay processes but which in fact originates from the decay signal of charged electron-hole complexes such as trions [19] (one electron and two holes or vice versa) or charged multi-excitons. Such additional contributions may inflate the apparent CM quantum yield (QY) or indeed appear to give evidence of CM where there is none (or where the true CM threshold is a lot greater than it appears). Califano [29] modelled the decay kinetics of biexcitons and both positive and negative trions in the InAs and InAs/CdSe core/shell systems and concluded for that material that trion decays, being several times slower than biexciton decays, should be distinguishable when fitting the bleach recovery signal. However, he also suggested that the large variability in reported CM QYs may also be at least partially explained by surface effects arising from different synthetic methods and surface treatments during sample purification and preparation. An early striking example of the realization that possible photocharging effects may complicate the extraction of CM parameters from experimental data comes from work by Pijpers et al. [30] on InAs QDs including InAs/CdSe/ZnSe core/shell/shell structures. In that work, a CM yield of 1.6 at an excitation energy of 2.7 E_g_ was reported. However, a subset of the same group of authors [31] revisited with TA measurements on the same system in the following year and with an improved approach to the extrapolation to zero excitation fluence failed to observe any CM up to excitation energies of 3.7 E_g_. Similarly, when Nair et al. attempted to observe CM in PbS, PbSe [26] and CdSe, CdTe [32] QDs using ultrafast TPL measurements, no or very limited evidence was found even above previously reported CM thresholds, that were well above the energy conservation limit. Time resolved photoluminescence experiments using time correlated photon counting as the detection method require only very weak excitation signals, thus ensuring that photocharging (e.g., originating from Auger recombination and ionization processes following multiple absorption events) is likely to be minimal or absent.

**Figure 3:**
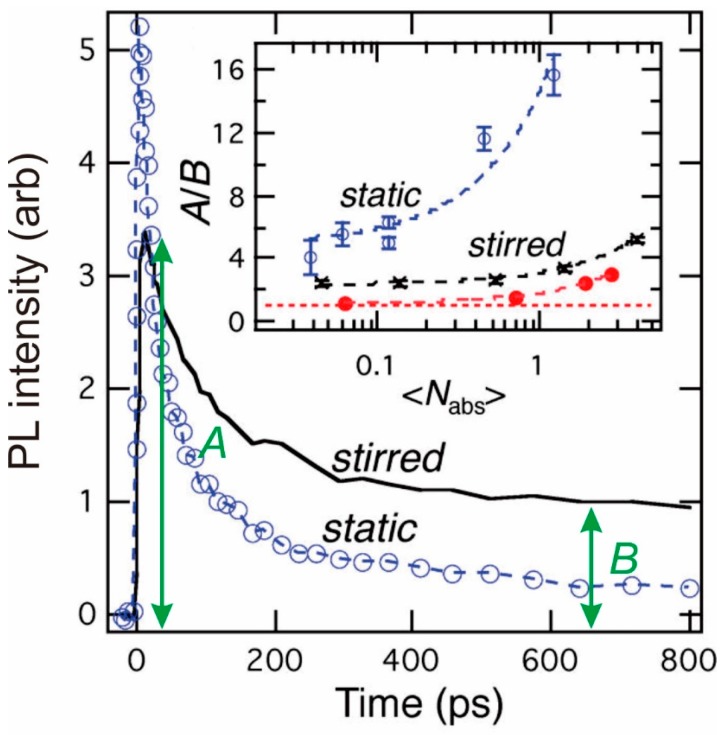
Effect of sample stirring on PL dynamics of PbSe QDs with significant static-stirred difference in PL dynamics (at exciton occupancy <N_abs_> = 1.4). This difference persists in the limit of low pump intensities. Inset: the ratio of the early to late-time PL signals (A/B) as a function of <N_abs_> for 3.08 eV (black crosses) and 1.54 eV (red circles) excitation. All data were acquired using femtosecond PL up-conversion with temporal resolution of ≤4 ps. Adapted with permission from ref. [33]. Copyright (2010) American Chemical Society.

McGuire et al. [33] considered the effect of a known degree of photocharging on the TA bleach signal and showed how the true CM QY could then be recovered from a measured signal (for PbSe QDs, see Figure 3). They also showed that the true CM signal could be obtained experimentally if the sample was stirred during measurements since any charged fraction of the measured solution would be swept out of the pump/probe beams and be diluted/have sufficient time to become neutralized by non-geminate recombination before re-entering the beam once more. Although only applicable to (low viscosity) solutions this measurement approach has now become commonplace. Other measures to combat photocharging include using a flow cell arrangement and also rastering the measurement cell through the beam, though this is perhaps more effective in terms of avoiding the runaway accumulation of damaged or precipitated material at the measurement cell interior surface with more fragile materials where colloid stability is marginal. For many materials in solution, stirring is sufficient both for the removal of photocharging artefacts and from the point of view of upholding the material stability. In the latter respect, monitoring the low excitation fluence steady state photoluminescence (PL) (single exciton) QY before and after TA, etc., measurements will give a clear indication of any incipient material damage during pump-probe measurements.

In further work, investigating the photocharging phenomenon in QDs, McGuire et al. [19] also showed how even low intensity steady state UV irradiation could lead to an accumulation of photocharged QDs. In their PbSe QD samples they estimated that there was a photochargeable fraction of 5–15% in steady state experiments. Whilst the probability of a single photon absorption event leading to photocharging was only 10^−3^–10^−4^, the long lifetime of charged species (tens of seconds) could lead to a significant build up (in the probe beam volume) unless this was offset by stirring. There has been some debate over whether photocharging affects the CM efficiency itself (as distinct from simply obscuring its measurement). McGuire et al. [19,33] suggest that in practice the presence of charges does not affect the actual CM efficiency whilst theoretical arguments (based on atomistic modelling of small PbSe clusters) have been advanced that suggest the CM efficiency might be suppressed under these circumstances by shifting the CM threshold to higher energies [34].

Not surprisingly, these findings cast a lot of doubt on some of the earlier reports of significant CM in QDs, and led to a spate of reassessment of several materials, the frequently studied PbSe QDs in particular. Trinh et al. [35] measured CM in PbSe taking steps to ensure proper discrimination between multi-excitons generated by CM and by multiple photon absorption by careful determination of the signal in the very low fluence range (where the average number of absorbed photons per QD could be as low as 0.05), and by accounting for any pump induced spectral shifts in their TA spectra by spectrally integrating over the whole bleach absorption peak rather than simply using the time dependent bleach intensity at a single wavelength at or in the vicinity of the peak. With this rigorous analysis, the authors found the CM efficiency to be almost a factor of two lower than in previous reports [36] for equivalent excitation energies of ~4.8 E_g_ but did confirm that CM *does* occur at such energies. Later TA measurements by Gdor et al. [37], again using spectral integration to ensure pump dependent peak shifts did not cloud the data analysis, showed no evidence of CM at excitation energies up to 3.7 E_g_ suggesting either a threshold in the range 3.7 E_g_–4.8 E_g_ (assuming that these studies used equivalent sized PbSe QDs) or that CM efficiencies and thresholds were batch dependent e.g., due to differences in surfaces or exposure to air, etc. Time resolved two photon photoemission spectroscopy measurements on PbSe thin films by Miaja-Avila et al. [38] allowed the hot electron relaxation and CM generation processes to be mapped. The samples were prepared as thin films and treated with ethanedithiol (EDT) after deposition, whilst measurements were carried out under high vacuum conditions. No evidence of CM was seen from intraband hot electrons with energies up to 3 E_g_, but for interband hot electrons CM was observed for energies of ~4 E_g_. This might be consistent with the differing findings in the Gdor et al. and Trinh et al. reports, but here again there is the scope for some debate as the EDT treatment was found by Beard et al. [39] to severely quench the CM response in their study of the effect of a range of surface treatments on PbSe films.

Ultrafast time resolved photoluminescence (TRPL) is often suggested as a potentially superior measurement technique for CM studies. Sub-nanosecond TRPL measurements using photon counting detectors and photon correlation generally only require very low fluence excitation (consistent with average numbers of absorbed photons per QD being <<0.1) and can offer good signal to noise ratios. However, when the bandgap and therefore detection wavelength move into the infrared (IR) which is beyond the silicon APD and photon counting photomultiplier ranges, detection becomes a problem. However, recent advances in detectors based on superconducting wires have shown the potential to greatly improve TRPL experiments in this energy range. The alternative in which photoluminescence photons are upconverted before detection with conventional lower wavelength range detectors (uPL) requires extremely long run durations (e.g., 10 h) to build up decay transients, whereas Sandberg et al. [40] reported being able to acquire signals between 10–100 times faster than with uPL. Using a combination of both techniques, they compared the electron-hole creation energies for both PbSe QDs and nanorods and concluded that the lower values for nanorods (2.6 E_g_) compared with QDs (3.2 E_g_) arises due to the elongated structure in the former. In the PbSe QDs and nanorods CM was observed at excitation energies in the 3 E_g_–5 E_g_ range.

## 3. Carrier Cooling and CM Efficiency

**Figure 4:**
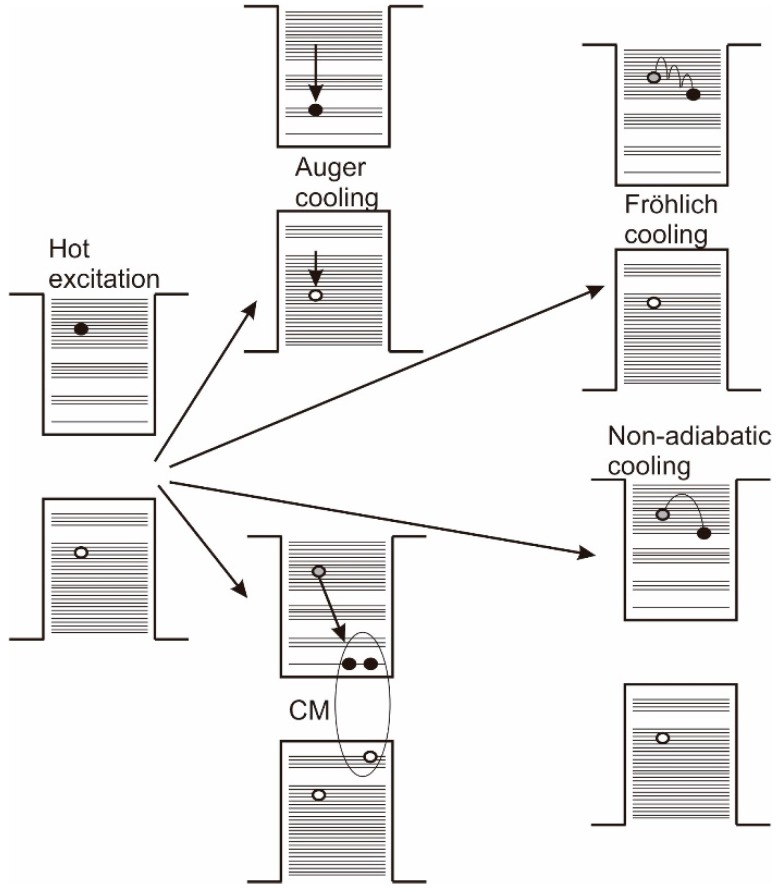
Possible relaxation channels following the absorption of a photon to create a hot exciton. These include electron-hole scattering where, if their effective masses are dissimilar, the (lighter) electron may lose energy to the (heavier) hole. The latter can then relax more readily by phonon emission through the denser hole states to the band edge. In the absence of the Auger cooling channel, excess energy may be lost by sequential phonon emission via a Fröhlich type relaxation. This should be very slow overall. Some groups have suggested that multiphonon emission steps may be possible in a non-adiabatic process, as a consequence of strong confinement or mediated by surface interactions (e.g., with ligand molecular vibrations). CM competes with all cooling channels, converting some or all of the excess energy into an additional (or at high excitation energies, several additional) exciton(s).

Following the excitation of a hot carrier, several possible fates may await. The carrier may relax to the band edge via either interband or intraband cooling processes (depending on how much excess energy above the bandgap the hot carrier has) and which will involve the emission of phonons and the eventual dissipation of excess energy to the lattice. Alternately the hot carrier may undergo CM resulting in the formation of two or more excitons (Figure 4). In order to select one or other material as good CM candidates, it is therefore useful to be able to compare the likely degree of competition between these two principal pathways by which the hot excited state relaxes. Stewart et al. [41,42] made ranking comparisons of the relative ratios of the electron hole creation energies of PbTe, PbSe, and PbS (1:1.8:4.5 respectively) predicted from their model for a competing two channel relaxation scheme and compared the trend with that seen for the measured 1P-1S cooling rate constants, k_1P1S_, PbTe:PbSe:PbS ≈ 1:2.0:4.2. Their analysis leads to the correlation that the electron hole creation energy, $\varepsilon_{eh}$, and cooling rate are connected as,

(1) $$\varepsilon_{eh}=\tau_{CM}k_{cool}$$

where the cooling rate $k_{cool}$ is assumed to scale for all three materials in proportion to the k_1P1S_ measured values. Thus, the CM rate, 1τCM is assumed to be broadly similar for all three materials and so the competing cooling channel dictates the $\varepsilon_{eh}$ trend. The cooling rate trend is similar, at least semi-quantitatively, to the trends in the bulk phonon cooling rates predicted from the product of the polar coupling constants, $\alpha_{F}$, and longitudinal optical (LO) phonon energies, $\hbar \omega_{LO}$, for the same materials. The Fröhlich coupling constant for bulk materials is given as,

(2) αF=(e2ℏ)(m2ℏωLO)12(1κ∞−1κ0)

where e is the electron charge, m is the effective mass, and $\kappa_{\infty }$, $\kappa_{0}$, are the high frequency and static dielectric constants respectively. The broad similarity in the CM rate is also reflected in the similarity for the biexciton Auger rate constants for the three materials. Whilst Auger recombination and CM can be considered as mutually inverse processes, their rates are not equal as the densities of the final states in either direction are not identical. However, Stewart et al. [42] also pointed out that the densities of states, $g_{xx}$, of the CM process that terminates in a biexciton, has a simple relationship to the Auger relaxation terminating in a hot exciton with a density of states (DOS), $g_{x}$, $g_{xx}\propto g_{x}^{n},\;n>2$. The authors [41] suggested that the trends across the three chalcogenides may point to a simple near linear scaling of the rates for the forward and reverse processes consistent with the DOS argument. Thus, measurement of Auger decay constants and calculation of cooling rates for the bulk materials would allow an estimate of the likely trends in CM efficiencies (at least for related materials where the approximations behind Equation (2) are broadly similar).

The hot carrier cooling process in QDs has been widely explored both experimentally and theoretically using a wide variety of modelling techniques. Kilina et al. [43] used an ab initio finite time domain model for 32 atom PbSe particles and observed evidence of multiphonon relaxation, strong coupling of both holes and electrons to acoustic phonon modes, and the relaxation of forbidden symmetry transitions, consistent with experimental findings. Hole relaxation only slightly outpaced electron relaxation with both occurring on the few picosecond timescales, whilst the model also failed to show any evidence of a phonon bottleneck that would otherwise dramatically slow carrier cooling.

Whilst the hole and electron effective masses in the lead chalcogenides are very similar, the situation is less symmetric in materials such as CdSe and InAs, where the electron effective mass is far smaller than that of the holes. Califano [44,45] considered the effect of an Auger cooling mechanism (see Figure 4) in a semi-empirical pseudopotential model whereby a hot electron interacts with a hole, losing energy and in the processes exciting the hole. The latter then efficiently cools through the more densely spaced valence bands, losing the energy just acquired from the interaction with the electron in this process and so increasing the overall cooling rate. Such Auger cooling channels could also effectively compete with the CM process (especially where the two carrier effective masses are very dissimilar) and account for more rapid cooling observed experimentally than anticipated for a multi-phonon cooling process. Using similar modelling techniques Califano [29] also calculated the InAs CM lifetime, $\tau_{CM}$, as a few tens of femtoseconds thus suggesting that CM should be able to compete effectively with phonon or Auger cooling channels.

A number of time domain ab initio modelling methods have been used by Neukirch and Prezhdo [46] to study not only exciton and biexciton formation but also to follow the relaxation processes such as carrier-phonon interactions and Auger cooling processes. These modelling methods are generally limited to relatively small structures of several tens of atoms at present due to limitations in processing speed and capacity, but nonetheless the results shed some light on many aspects of experimental findings with much larger particles. In regard to cooling mechanisms the modelling approaches considered various aspects of electron-phonon coupling, both direct and mediated by surface ligands. In both CdSe and PbSe alike, direct electron-phonon and hole-phonon coupling was seen to involve predominantly lower frequency acoustic modes rather than high frequency optical modes as in bulk materials. These processes also compete with Auger type cooling channels. The latter are assumed to be less effective in Pb chalcogenides than CdSe for example, on account of the very symmetric conduction and valence bands in the former and their very small effective masses for both holes and electrons. This means that electron-hole interactions result in very little energy transfer and little advantage in dissipating the excess energy. In CdSe by comparison, the hotter electrons can short circuit the need to cool by direct phonon emission through more sparsely separated bands by transferring energy to heavier holes which can then relax more efficiently by phonon emission through more densely spaced bands. However, electron cooling could be enhanced (in either type of material) by interaction with high energy vibrational modes due to surface ligands. The authors point out that separation of the electrons and holes in materials such as CdSe (e.g., in heterostructures) could suppress the extent of electron-hole energy transfer and allow CM the chance to compete more effectively with cooling.

The intraband cooling rates in PbSe and CdSe QDs and their dependence on temperature and environmental modifications (ligand and solvent changes) have been compared by Schaller et al. [47]. They concluded that in both materials, the influence of the surface on the cooling rates was negligible [47,48]. In CdSe QDs the cooling rate was thermally insensitive whereas PbSe QDs showed a size dependent activation of a cooling channel (e.g., above 130 K and 170 K for 3.5 nm and 1.9 nm radii QDs respectively). The conclusion was that in CdSe QDs the Auger cooling channel was the dominant process whilst in PbSe QDs the latter was ineffective with a phonon relaxation channel being more important. Since a single phonon process would not be sufficiently fast enough to account for the cooling rates involved, a non-adiabatically coupled multiphonon emission process was suggested.

Ten Cate et al. [49] studied the CM efficiency and cooling rate dependence on temperature in EDT linked PbSe solid films infilled with Al_2_O_3_ or Al_2_O_3_/ZnO using the TRMC technique. Over the range 90 K–295 K there was no temperature dependence for either cooling rates or CM suggesting that phonons are not required or do not participate in the matching of transitions in the CM process. The efficiency was then limited by the cooling/CM competition, determined by spontaneous LO phonon emission. Interestingly the oxide infilling was found to be necessary to allow the CM process to be observed [27]. Non-infilled PbSe/EDT films showed no significant CM response in agreement with other similar studies [38,39].

The size dependence of the carrier cooling rates has been determined by several groups. Stolle et al. [50] saw a decline in the overall carrier cooling rate with increasing diameter in CuInSe_2_ QDs consistent with a linear dependence on volume. Harbold et al. [51] saw a similar rising trend in the intraband relaxation times with PbSe QD diameters which may be consistent with a similar volume scaling law for the cooling rate. In the latter case, symmetrical valence and conduction bands mean that differences in hole and electron cooling rates cannot readily be distinguished. The decrease in cooling rates with increasing size in the face of a decreasing spacing of intraband states may suggest that cooling by LO phonon emission (as in bulk materials) is not the dominant cooling mechanism [52] in such QDs. Cooling dynamics have also been studied in InAs QDs which were either treated with pyridine as a surface species and measurements made in the presence of a sodium biphenyl reducing agent, or as regular trioctylphosphine oxide capped QDs under double pump pulse (IR + visible) excitation. In this case, the electron effective mass is far lower than that of the hole, but by removing the latter via the chemical treatments or by separating the charges, the electron cooling time in the absence of the hole was determined to be around an order of magnitude slower than when both types of carriers were present. This also reinforces the fact that in QDs, especially those with disparate carrier effective masses, carrier-carrier scattering effects play a very strong role in the cooling process.

**Figure 5:**
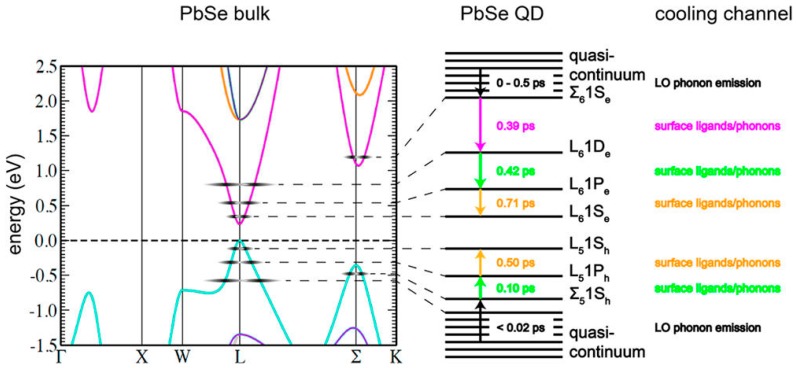
Schematic model of the PbSe QD electronic structure, alongside the corresponding bulk band structure (left), with cooling time constants for transitions between successive energy levels. With kind permission of the ACS; the original Open Access article [54], published under a Creative Commons License can be found at http://pubs.acs.org/doi/pdfplus/10.1021/acsnano.7b02506. For permissions to make further re-use of this figure please consult the ACS at http://pubs.acs.org/page/rightslinkno.jsp.

The individual cooling rates of electrons and holes in PbSe QDs have been determined by Spoor et al. [53,54] (see Figure 5) where the separation of hole and electron transitions was facilitated by using a dye (methylene blue) to extract photogenerated electrons and so identify which transitions in TA spectra arise from either type of carrier relaxation. The work was further extended to higher excited state relaxations away from the band edge [54]. Near the band gap, the electron and hole cooling rates were 0.54 eV/ps and 2.75 eV/ps respectively whilst for the highest excited states the rates increased to 1.52 eV/ps and 6.8 eV/ps. Given the symmetries in the electron and hole bands near the band gap and the similarities in the two effective masses the band edge cooling rates might be expected to be closer, though the differences in the higher excited state cooling rates could be attributed to less symmetric band structures well away from the band edge. The near band edge dissimilarity in cooling rates does lend support to Zunger et al.’s [55] modelling of PbSe QDs which shows differing valence and conduction band DOS (with the valence band DOS being greater than for the conduction band) in contrast to the case for the bulk material. Spoor et al. [54] attributed only the cooling at very high excess energies for either carrier to be due to bulk-like LO phonon emission but at lower energies resolved several separate cooling steps (in cooling rates) which were each explained as involving phonon or surface ligand vibrational modes in view of the larger separations in energy levels nearer the band edge (Figure 5).

Direct observation of electron-phonon interactions and the discrimination between coherent optical and acoustic phonon mode couplings in CdSe QDs have been reported by Kambhampati et al. [56,57] using state resolved transient spectroscopy. They reported that the acoustic phonon coupling is slightly stronger than that for the exciton-optical phonon interaction.

## 4. CM Mechanisms

So far little has been said about the exact nature of the CM process or mechanism, with the foregoing only considering the competition between CM (by whatever means) and other cooling processes. CM is well known in bulk semiconductors and is described by a number of impact ionization variants based on the different types of band structure encountered near the band gap. Expressions are given for the threshold energies for a number of these cases by Landsberg [1], for example. The threshold energies for those semiconductors (e.g., Si and Ge), for a long time considered the most relevant for solar energy generation, are unfortunately too high (≥4 E_g_)to be of practical use in generating additional carriers with the terrestrial solar spectrum [9,10]. One of the primary uses of impact ionization in bulk semiconductors has been in the design and manufacture of solid state APDs [2] with photocurrent gain typically in the ×10–×100 range. Here kinetic energy for ionization is supplied by an accelerating bias voltage across the junction rather than by photogenerated hot carriers but otherwise the multiplication process is identical. The range of materials used in APDs includes Si, Ge, GaAs, GaP, InP, InAs, InSb, and InGaAs, GaAlSb and HgCdTe alloys. In materials such as GaAlSb and HgCdTe which have zincblende structures, spin-orbit coupling is exhibited with the split-off valence band lying below the band gap by an energy interval Δ. When the latter is on resonance with the bandgap energy E_g_, the impact ionization process can be enhanced and since the inverse Auger transitions are all vertical (no changes in momentum), the energy threshold is in principle simply 2 E_g_ However, this circumstance has to be contrived by adjusting the alloy compositions which then leads to E_g_ values that are too low for practical solar energy applications, but still useful for IR photodetectors.

The initial expectation was that CM in QDs should be both more efficient and have a lower threshold than in the corresponding bulk materials owing to the strong Coulomb interactions in strongly confined QDs [7]. Califano et al. [58] formulated a model based on impact ionization as the basis for direct carrier multiplication (DCM), and indeed saw higher multiplication rates in CdSe QDs than in bulk. Their pseudopotential model found the AR and DCM processes to be highly sensitive to the QD surface whilst the presence of gaps in the hole manifold of states encountered for excess energies well above threshold, allowed Auger cooling the chance to compete strongly with DCM across ranges corresponding to the gaps. Separation of the electrons and holes (e.g., in heterostructures) to prevent Auger cooling was a remedy suggested to suppress this competition. The same modelling approach was extended to PbSe QDs [59] and again only impact ionization was found to be necessary as the basic mechanism for CM and to produce a model which replicated the experimental findings of the time in terms of low threshold energies (2.2 E_g_) and sub ps CM rates. Many groups revised up their threshold energy values later on, in the light of the above-mentioned controversies over the appearance of CM-like artefacts in early measurements. More recently, Califano [44] examined the DCM and Auger cooling competition in CdSe QDs using both an impact ionization and a CM model-independent approach and found close agreement between both theoretical approaches.

**Figure 6:**
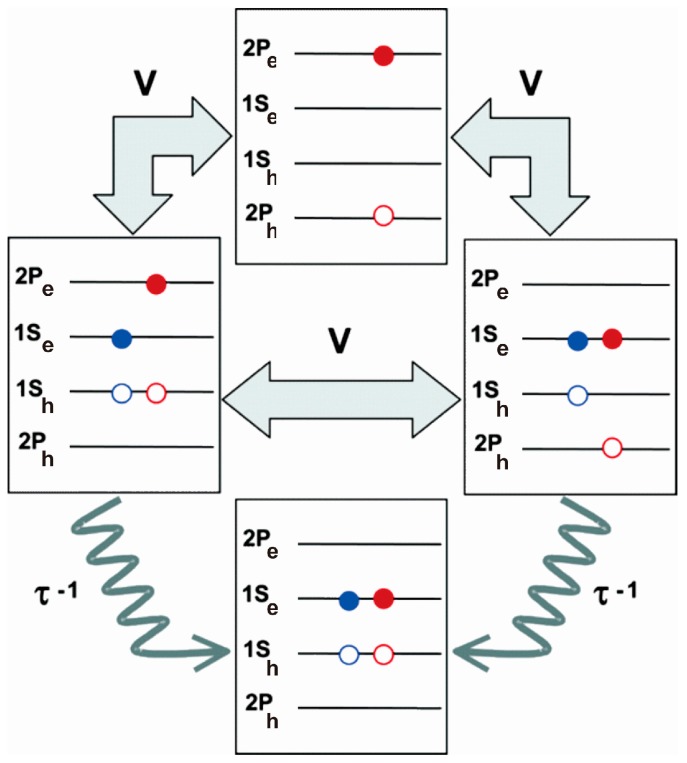
Photoexcitation at 3 E_g_ creates a 2P_e_-2P_h_ exciton state. This state is coupled to multiparticle states with matrix element V and forms a coherent superposition of single and multiparticle exciton states within ~250 fs. The coherent superposition dephases due to interactions with phonons; asymmetric states (such as a 2P_e_-1S_h_) couple strongly to longitudinal optical (LO) phonons and dephase at a rate of *τ*^−1^. Adapted with permission from ref. [9]. Copyright (2005) American Chemical Society.

Low threshold energies (of around 2 E_g_) found experimentally in PbSe and PbS prompted Ellingson et al. [9] to suggest a more elaborate CM mechanism, as depicted in Figure 6, by which multi-excitons are formed via a coherent superposition of single and multiexciton states, with the final outcome (multiexcitons) dictated by faster phonon relaxation (dephasing) from the multiexcitons than the hot single exciton. However, the lack of any observation of oscillations between single exciton and multiexciton states before dephasing could complete prompted Klimov [28,60], to propose an alternate CM mechanism where multiexcitons are generated directly via Coulomb coupling to a hot virtual (rather than real) single exciton state. The coherent superposition model has also been explored extensively by Shabaev et al. [18,61]. Again, oscillation (sometimes termed quantum beats) between the (hot) single exciton and multiexciton states is predicted for strongly coupled systems where the multiexciton decay rate is much slower than the Coulomb driven coupling rate between the two states. However, even theoretically the authors show that such oscillations can be obscured due to the large multiplicity of multiexciton states [18]. Shabaev et al. [18] also pointed out that, in contrast to the case in spherical QDs, in nanorods, nanowires and 2D nanoparticles there can be significant and unscreened penetration of the hole and electron electric fields into the surrounding and usually markedly lower permittivity dielectric surrounding the nanoparticle. This tends to strongly increase the rate of the Coulomb coupling via an enhanced exciton binding energy. Provided that the multiexciton relaxation (dephasing) is fast enough, this should result in enhanced CM efficiencies and lower thresholds in such materials.

Schaller et al. [62] gave a simple expression for the threshold energy, *E_th_*, (irrespective of the details of the CM mechanism) on the basis of simple carrier effective mass considerations as,

(3) Eth=(2+memh)Eg

**Figure 7:**
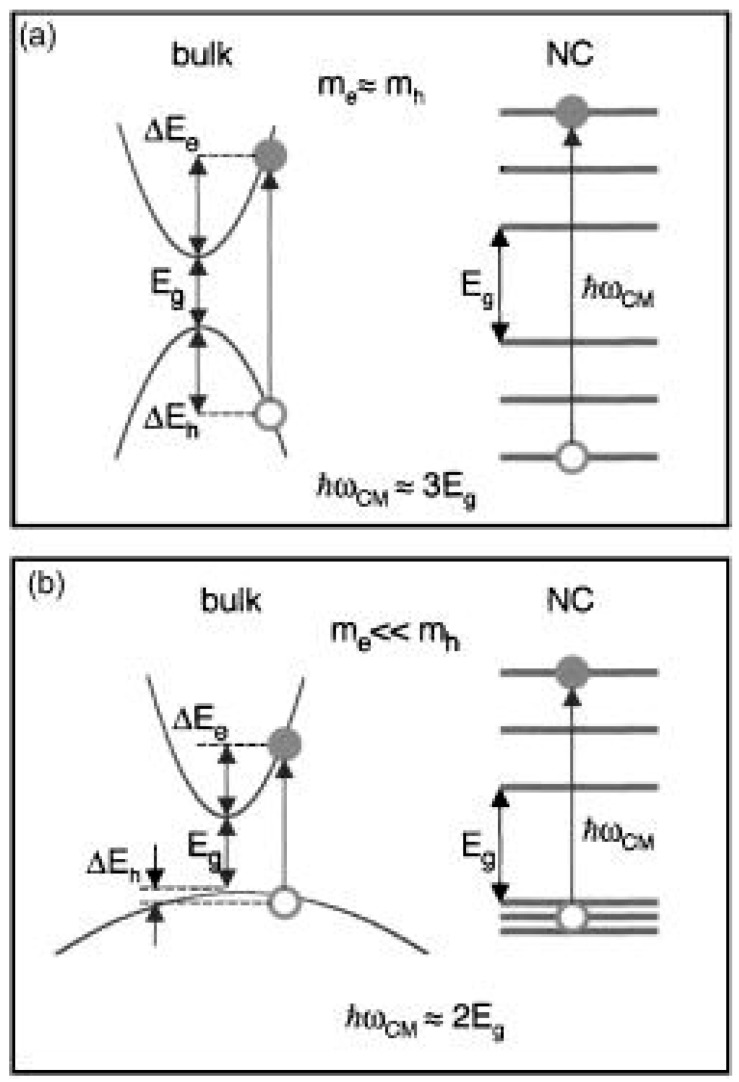
(**a**) Bulk-like, carrier effective-mass considerations indicate that in the case of similar electron and hole masses m_e_ ≈ m_h_ the onset of CM is ~3 E_g_, whereas it approaches 2 E_g_ (**b**) in the case for which the electron and hole masses are very dissimilar m_e_ << m_h_. Reprinted from ref. [62], with the permission of AIP Publishing.

On this basis semiconductors, such as the lead chalcogenides, which have almost equal hole and electron effective masses are expected to have thresholds near 3 E_g_. CdSe with m_e_/m_h_ at 0.17 should have a threshold at 2.17 E_g_ whilst materials such as Hg_1−*x*_Cd_*x*_Te, and the III–V materials InSb and InAs where the electron effective mass can be far lighter than that of the hole would have thresholds approaching the energy conservation limit of 2 E_g_ (Figure 7). Schaller et al. [63] also suggested that if the biexciton Coulomb interaction energy ($\Delta_{XX}$) is sufficiently strong, the threshold energy could be lowered even below the 2 E_g_ energy conservation limit,

(4) $$E_{th}=2E_{g}-\left|\Delta_{XX}\right|$$

This situation could arise in the low bandgap, lower permittivity, III–V materials such as InAs and InSb, whereas the lead chalcogenides have much higher permittivities, correspondingly stronger Coulomb interactions and so lower biexciton energies. Even in CdSe, neutral biexciton binding energies may reach several tens of meV [64]. It was also suggested that even larger attractive biexciton binding energies could be specifically engineered in heterostructures [64,65].

**Figure 8:**
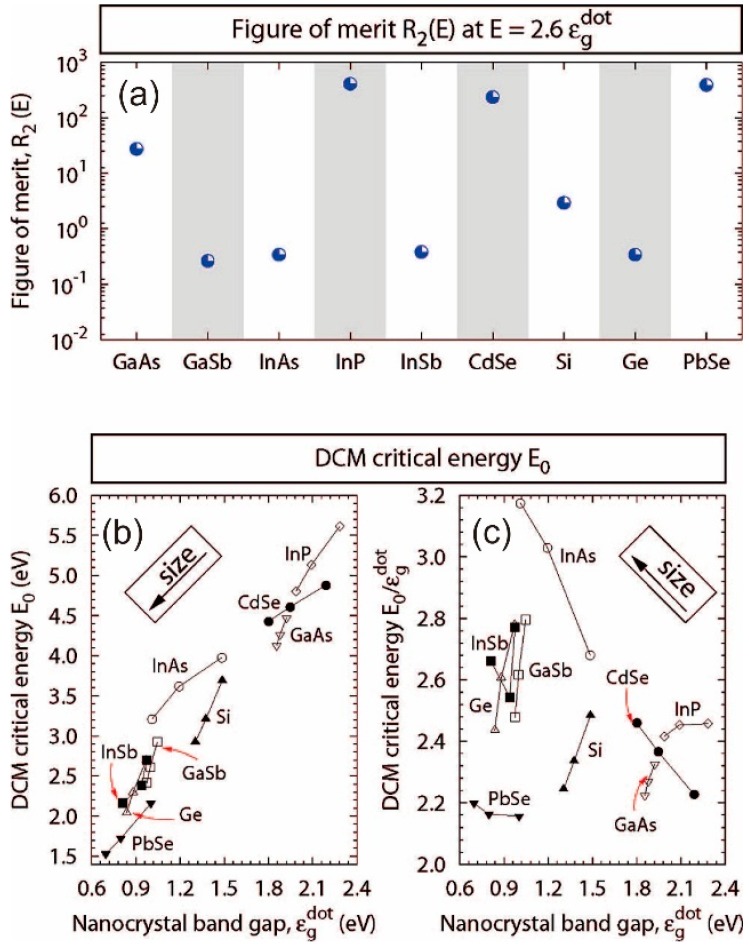
(**a**) The direct carrier multiplication (DCM) figure of merit R_2_(E) at photon energy E = 2.6 $\varepsilon_{g}^{dot}$ for different nanocrystals of size 6 × 6 × 6 unit cells; (**b**,**c**), The DCM critical energy E_0_, i.e., the photon energy at which R_2_(E) = 1, is shown as a function of nanocrystal band gap. In (**b**) E_0_ is shown in absolute units (eV), while in (**c**) it is normalized as E_0_/$\varepsilon_{g}^{dot}$. For each material three points corresponding to three sizes are shown (smaller, larger and at the nanocrystal gap). Part (**b**) shows that as the dot size increases E_0_ decreases, whereas part (**c**) shows that the normalized E_0_/$\varepsilon_{g}^{dot}$ sometimes increases (e.g., Si) and sometimes decreases (e.g., InAs) as the dot size increases. Adapted with permission from ref. [66]. Copyright (2008) American Chemical Society.

An alternate approach to the modelling of CM was adopted by Luo et al. [66], in particular for the purposes of surveying a wide range of materials based on their bulk electronic parameters to compare thresholds and efficiencies in QD forms. Rather than adopting the coherent superposition of states or virtual exciton mediated mechanisms, they simply considered the same impact ionization mechanism found in bulk materials, finding it sufficient to account for many experimentally observed measurements of thresholds and efficiencies, although phonon relaxation and the influence of surface states are not explicitly covered. A key figure of merit for CM, R_2_(E), was deemed to be based on the ratio of the Coulombically coupled biexciton density of states and the exciton density of states. As such it represents the extent to which the CM process is favoured over the inverse process, Auger recombination. Figure 8a shows a comparison of R_2_(E) values for a number of materials, and interestingly CdSe and PbSe are shown to be relatively similar. Figure 8b,c shows the calculated threshold data both in absolute energy units and also in terms of QD bandgap normalized values.

**Figure 9:**
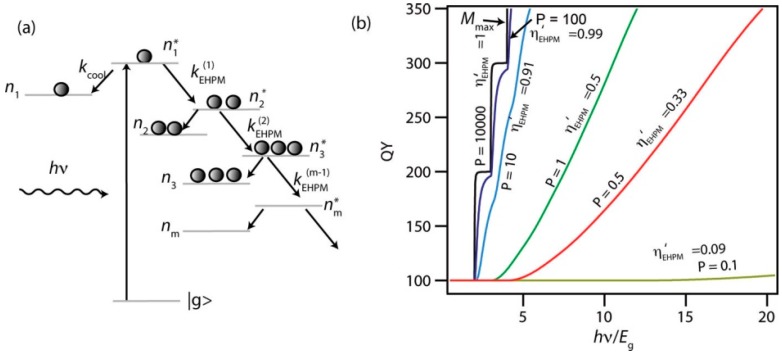
(**a**) Cascade scheme for electron hole pair multiplication (EHPM) considered by Beard et al. [11]. A high-energy photon creates an exciton with excess energy, n^*^_1_. The hot exciton can lose energy by cooling or multiplication to form either n_1_ or a hot biexciton, n^*^_2_ and so on. (**b**) Calculated exciton QYs for different values of *P*, *η*′_EHPM_ is shown at each *P* value. Reprinted with permission from ref. [11]. Copyright (2010) American Chemical Society.

The competition between CM and carrier cooling processes was addressed by Beard et al. [11] who used a coupled rate equation approach to model a cascaded set of relaxation processes such as depicted in Figure 9a. For the cooling rates, $k_{cool}$, they employed a parameterization due to Ridley [67] for bulk materials which is linked to the rate for each electron hole pair multiplication (EHPM) cascade step, $k_{EHPM}$,

(5) $$k_{EHPM}=k_{cool}P{\left(\frac{E-E_{th}}{E_{th}}\right)}^{s}$$

where *P* and *s* are considered as adjustable parameters. This then allowed the authors to calculate the net CM rate for given values of *P* and *s* using a Monte Carlo method (see Figure 9b).

Beard et al. [11] also compared experimental QD and bulk CM QY data, each in terms of bandgap normalized photon energy, showing that in this format QDs have lower thresholds than their bulk counterparts, and steeper QY vs. normalized energy gradients above the respective thresholds.

Tight binding methods have also been used as the basis for theoretical models of CM. Tight binding is used to determine the band structure and then transition rates (*W*) for processes such as impact ionization can be calculated using the Fermi golden rule expression for each of the transitions allowed between the exciton and multiexciton manifolds:

(6) $$W=2\pi {\left|V_{if}\right|}^{2}\frac{\rho_{f}\left(E_{i}\right)}{\hbar }$$

Here $\rho_{f}\left(E_{i}\right)$ is the density of final (multiexciton) states at the energy of the initial state, and $\left|V_{if}\right|$ is the transition matrix element of the Coulomb interaction between initial and final states. Allan and Delerue, together with others, have applied this approach both to the calculation of IR absorption spectra of HgTe QDs [68,69,70], (along with experimental comparisons) and also to the calculation of CM rates in a wide range of QD materials such as PbS [71], PbSe [71,72,73], InAs [72], Si [72], Sn [74] and HgTe [69]. In these studies Allan and Delerue found that impact ionization was a sufficient mechanism to explain all but the highest CM QYs, but for the very high exciton multiplicities initially reported for PbSe and InAs they found that an alternate or additional CM channel might be required (e.g., [71]) In their study of InAs, Si and PbSe QDs [72] they compared both impact ionization and multiexciton/exciton superposition of states models, but still found that very high multiplication factors (>×5) were hard to reconcile with the very low densities of final multiexciton states at such high excitation energies. However, the revisions of experimental data along the lines discussed earlier (Section 2), have probably helped somewhat to bring theory and experiment more into line once more [73].

Atomistic semiempirical pseudopotential methods have also been used to calculate densities of states, followed by transition rate evaluation by applying the Fermi golden rule. Rabani and Baer [75,76] used this approach with the inclusion of phonon interactions to model CM rates, again in Si, InAs and CdSe QDs of various sizes with either pseudohydrogen atom passivation or assuming ligand passivation on surface metal atoms. The InAs and CdSe QDs were close to stoichiometric in composition which may be a little unrealistic for larger colloidal QDs that are probably slightly more metal rich in practice [77] where ligands that bind to metal atoms are used in the syntheses. Their CM mechanism involved the decay of either a hot hole or hot electron to form respectively a positively or negatively charged trion (with the counter charge as a spectator), hence their need to compute the corresponding trion densities of states. With lower trion densities of states in InAs, CM is predicted to be weaker in the latter than in Si and CdSe. Small QDs were shown to have greater CM rates (on an E_g_ normalized energy scale) than larger dots. The electronic band structure of InSb QDs has also been explored by Sills et al. [78]. Here the relaxation of the biexciton state is known to be extraordinarily fast [79] (6 ps–16 ps rather than several tens of ps) prompting debate over whether this is due to Auger recombination or some other process. One feature of the Sills et al. study [78] is that although they again used pseudohydrogen passivation, they also constructed and compared QDs that were either metal rich or pnictogen rich and saw differences in the behaviour of the conduction band variation with QD size according to the stoichiometry.

The three principal CM mechanisms: impact ionization, coherent superposition of exciton and multiexciton states and direct multiexciton formation via a virtual exciton state have been compared by Neukirch and Prezhdo [46] alongside their ab initio modelling methods for small PbSe [43], CdSe, Si and Ge QDs. Such methods are atomistic in nature and may include descriptions of surface ligands (though often simple pseudohydrogen passivation is chosen), defects and dopants and the inclusion of carrier phonon interactions. Limitations still include the size of the system that can be handled (typically a few up to 70 or so metal atoms) and often studies are limited to (totally or nearly) stoichiometric compositions for binary QDs. Theoretical models and CM mechanisms are also comprehensively described in the recent Chemical Review by Pietryga et al. [3].

## 5. Engineering Nanostructures to Influence the CM/Cooling Competition

### 5.1. Size Effects

**Figure 10:**
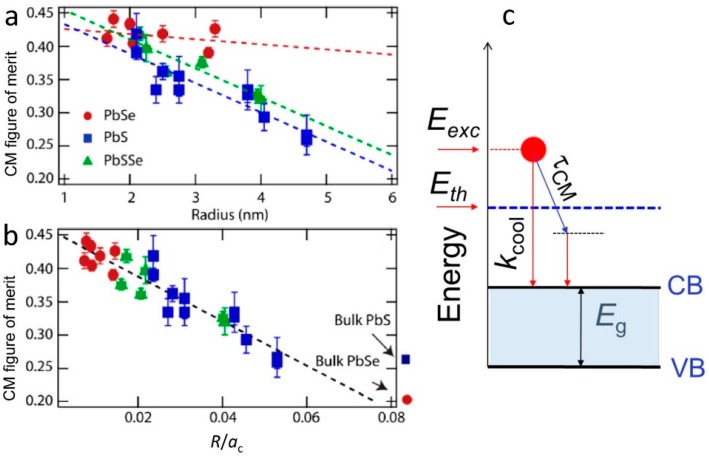
(**a**) Plot of the CM figure of merit for PbSe (red circles), PbS (blue squares), and PbSe_*x*_S_1−*x*_ (green triangles) QD as a function of QD radius; (**b**) Same data as in (**a**) plotted as a function of R/a_c_; (**c**) Main features of the “Window-of-Opportunity” model, where the overall CM yield is determined by the competition between non-CM carrier cooling (*k_cool_*) and the intrinsic time scale of CM (*τ_CM_*). CM processes may occur within the time window when the energy of the carrier remains above the energetic threshold for CM (*E_th_*), which is determined by the excess energy of the excitation (*E_exc_*) and the rate of non-CM relaxation (*k_cool_*). Reprinted with permission from ref. [3]. Copyright (2016) American Chemical Society.

The size dependence for biexciton recombination (AR) in QDs has long been established to show an inverse volume scaling dependence [80]. The CM lifetime, with CM being considered as the inverse of the AR process, is expected to show the same size dependence. However, as different final densities of states are involved in the two processes, so CM lifetimes are proportionately faster (sub-ps rather than tens of ps). It is difficult to directly measure CM lifetimes, partly due to the short experimental timescale, but also due to the fact that CM will always be in competition with other cooling processes that will obscure the measurement. The size dependence of the intraband cooling process is known [47] to show a rapid increase with decreasing QD diameter, which should increase (scaled) threshold energies and reduce the CM efficiency above the threshold for a given CM lifetime. The overall balance between the two competing processes will determine the size dependence of the CM threshold and efficiency. For PbS, PbSe and PbS*_x_*Se_1−*x*_ alloys Midgett et al. [81] report different dependences of the CM figure of merit vs. QD radius that show correlation with the degree of confinement for each material. In PbSe QDs the confinement was the strongest and very little size dependence was observed over the range of sizes studied. For PbS and the alloy QDs the size dependence was more marked with a linear trend vs. QD size. All three sets of data were reconciled to a single linear curve when presented as a function of the particle diameter normalized by a critical QD size, a_c_, defined as the size at which the electron-hole interaction energy and the confinement energies were equal [3,81] (Figure 10).

El-Ballouli et al. [82] presented their data on CM in PbS QDs in a different format, showing the CM QYs vs. QD bandgap energy where each different sized QD was excited at 3 E_g_ in each case. The study was similar to earlier work on CM and intraband carrier relaxation rates in PbS QDs by Nootz et al. [83] with cooling rates ranging from approximately 0.06 eV/ps–2 eV/ps over a range of QD sizes (1.4 nm–4.5 nm). Comparison with similar literature data for PbSe QDs [47] showed a very similar cooling rate trend. Schaller et al. [47] pointed out that the similar hole and electron effective masses in lead chalcogenide QDs rules out competing Auger cooling via the transfer of excess energy from electrons to holes. This would mean that cooling measurements should only be sensitive to phonon mediated relaxation channels. Contrastingly, in QDs with dissimilar carrier effective masses this is not the case. Cooling rates in CdSe for example are slightly higher than for PbSe [84] though the size dependence does follow a similar but displaced trend. By way of comparison, hot carrier cooling rates have also been measured in Si QDs by Bergren et al. [85] using terahertz transient spectroscopy methods where hot carrier lifetimes in the range of 500 fs–900 fs for QDs in the 1 nm–4 nm radius range were observed.

The perils of including photocharging artefacts in CM measurements are nowadays well understood, and can usually be obviated by using stirred or flowing samples for solution measurements or corrected for following for example Nootz et al.’s approach [83]. Padilha et al. [86] pointed out in their study of (intentional) photocharging in QDs, that the latter process is also itself size dependent and so may therefore complicate apparent CM size dependence if not properly factored out. They reported that photocharging in PbSe QDs had an onset between 2.5 E_g_ and 3 E_g_ (similar to many reported CM thresholds for this material) and may itself be connected with the presence of multiexcitons (from CM or multiple photon absorption) driving an Auger ionization process. The size dependence for photocharging may therefore mask the underlying CM size variation if not properly suppressed or accounted for.

### 5.2. Shape and Dimensionality Effects

**Figure 11:**
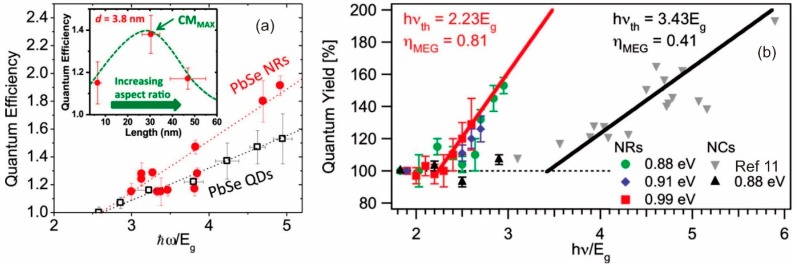
Comparisons of PbSe nanorod and QD CM performance: (**a**) QY threshold curves reported by Padilha et al. [21]. Inset showing evidence of an optimum aspect ratio for 3.8 nm diameter nanorods; (**b**) A similar comparison from Cunningham et al. [90] showing different CM onsets when comparing nanorod performance to literature QD data [11]. (**a**) Reprinted with permission from ref. [21]. Copyright (2013) American Chemical Society. (**b**) Reprinted with permission from ref. [90]. Copyright (2011) American Chemical Society.

It was recognized quite early that elongated nanoparticles such as nanorods and nanowires would differ from QDs in terms of both the strength of carrier-carrier interactions (e.g., exciton binding strengths) and potentially carrier cooling rates [52]. Yu et al. [52] found faster relaxation in thin CdSe nanorods than thicker ones of comparable length, pointing to the importance of the nanorod aspect ratio. Although their study did not explore CM, they concluded that Auger cooling via electron-hole interactions could be playing a strong role in the cooling process, rather than LO phonon emission, as there would have been a greater density of states in the thin nanorod case compared with the thicker nanorods. In CdSe the electron and hole effective masses differ, so electrons may efficiently cool by transferring their excess energy to holes. However, if the latter was the dominant cooling process, it should be sensitive to the removal of holes by the addition of hole scavengers, etc., [87]. Whilst hole removal did affect the cooling rate, cooling could not be prevented completely, pointing to other cooling channels operating in parallel with electron-hole scattering. In materials such as PbSe, the electron and hole effective masses are almost equal, resulting in little Auger cooling benefit from electron hole-scattering. Bartnik et al. [88] used a multiband model to calculate electronic structures in PbSe nanorods and nanowires and predicted a strong enhancement in Coulomb interaction strengths that should enhance both Auger non-radiative recombination and conversely CM rates. A large part of the enhancement was found to arise from the strong dielectric contrast between the semiconductor and the surrounding medium, which is not significantly screened as in the case of spherical QDs [18]. The dielectric constant of semiconductor materials such as PbSe, etc., can be an order of magnitude greater than surrounding media such as organic solvents, ligands, etc. Padilha et al. [21,41,89], reported a decrease in the Auger recombination rate in PbSe nanorods rather than an increase, but yet saw that the CM rate was higher in nanorods. Moreover, they saw an optimum aspect ratio (length: width between 6:1 and 7:1) beyond which the CM enhancement declined, eventually reaching a point where the nanorods performed more poorly than equivalent QDs (Figure 11). The initial contradiction between Auger recombination and CM rates was attributed to the very strong exciton binding in 1-D structures which changes the nature of the Auger recombination process from a three particle interaction (i.e., charged trions) in QDs to a two particle (neutral exciton-exciton) process in nanorods [21,89]. At high aspects ratios, the decline in CM was attributed to the resumption of bulk-like momentum conservation constraints.

**Figure 12:**
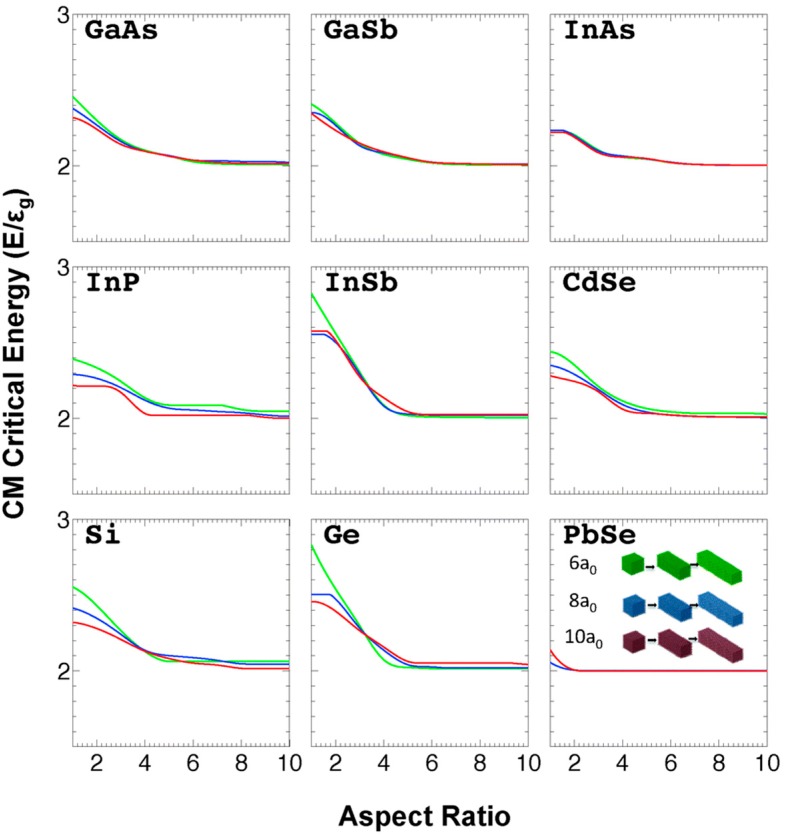
Variation of (normalized) CM critical energies for different materials as a function of aspect ratio at constant volume, calculated for three different volumes: 6a_0_^3^, 8a_0_^3^ and 10a_0_^3^. Reprinted by permission from Macmillan Publishers Ltd.: Physical Chemistry Chemical Physics ref. [91], copyright (2015).

An effective mass model has been used by Sills and Califano [91] to mount a comparison of the CM figures of merit vs. aspect ratio for a wide range of semiconductors (GaAs, GaSb, InAs, InP, InSb, CdSe, Ge, Si and PbSe). A simple rectangular cross section rod was assumed and the influence of shape factors on the electronic structure (DOS) without including the effect of varying Coulomb interactions and surface effects was investigated. Whilst the initial drop in bandgap energy normalized CM thresholds with increasing aspect ratio was observed, at large aspect ratios the materials each tended towards thresholds corresponding to energy conservation alone (i.e., *E_th_* = 2 E_g_) whereas the neglected strong Coulomb interactions will probably modify this behaviour somewhat (Figure 12).

**Figure 13:**
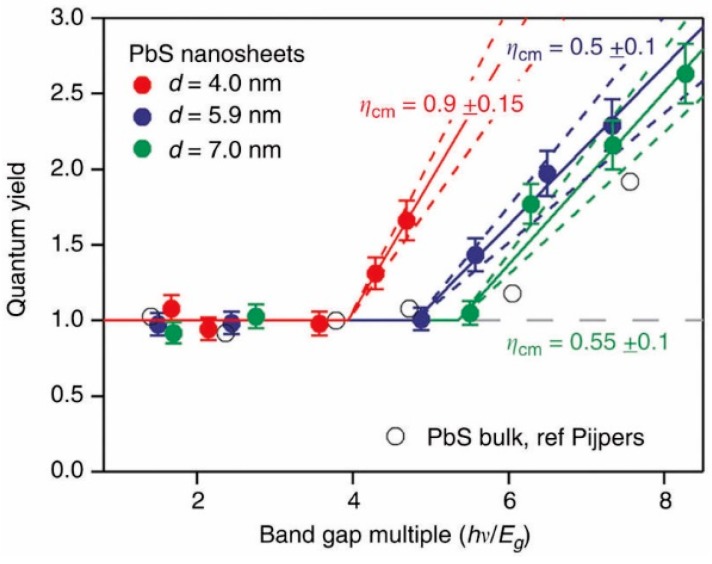
Quantum yield plotted vs. band gap multiple *hν*/E_g_, for PbS nanosheets with thicknesses, d, as indicated. In addition, literature data on obtained quantum yields in PbS bulk are shown [99]. The slope of the data points is equal to the CM efficiency *η*_cm_ (solid lines). Adapted by permission from Macmillan Publishers Ltd.: Nature Communications ref. [97], copyright (2014).

The observation about the sensitivity of nanorods to the surrounding dielectric constant also applies to thin 2D nanosheet materials (nanoplatelets) as well [18]. Compared with QDs and nanorods, the Coulomb interaction strength in nanoplatelets should be higher, whilst the electronic density of states should be higher than that in nanorods [92]. However, as in longer nanorods, 2D structures that are thicker and more extended in their lateral two dimensions will tend towards the bulk regarding momentum conservation requirements for CM [3]. A number of groups have synthesized such materials both by direct methods [93], and by subsequent ion exchange [94,95,96], and a few have investigated their Auger recombination rates, energy transfer and CM QYs. Aerts et al. [97] synthesized PbS nanosheets (with micron lateral dimensions, but thicknesses of a few nm) and for the thinnest sheets (4 nm) observed a lowering of the normalized CM threshold relative to bulk material, an increase in the CM efficiency, and a thickness dependent bandgap blue shift due to the layer confinement (Figure 13). In CdSe and CdSe/CdS/ZnS core/shell nanoplatelets, biexciton Auger decay rates of 0.07 ns^−1^ and 0.12 ns^−1^ were measured by Kunneman et al. [22] using TRPL techniques. Again, the fact that the Auger rate is about an order of magnitude slower than for QDs or nanorods of comparable volume is attributed to the necessity for some degree of adherence to momentum conservation rules in nanoplatelets. Slower Auger recombination was exploited by Rowland et al. [98] in the use of much faster Förster resonant energy transfer (FRET, transfer time 6 ps–23 ps) to drive the transfer of excitations between nanoplatelets of two differing sizes, optimized so that one was spectrally positioned to act as a donor and the other as the acceptor for FRET. PbSe_1−*x*_S*_x_* nanoplatelets derived from CdSe_1−*x*_S*_x_* nanocrystals by interparticle attachment followed by cation exchange have also been observed to show long (from 70 ps to 80 ps) decay times and shown to offer advantages in photoelectrochemical hydrogen generation [94].

### 5.3. Heterostructures

A number of different QD heterostructures have been explored for possible enhancement of CM performance. Several early studies used type I core/shells [64,100] (e.g., CdSe/ZnS), though the benefits of this type of band alignment for the CM rate or for carrier cooling rates is not thought to be so significant. Indeed, the Auger recombination rate in type I QDs is believed to scale with the volume of the whole structure, just as for core only QDs [101,102]. There may be some minor benefit from improved surface passivation and therefore reduced carrier trapping at the surface [103]; even recently Singhal et al. [104] compared CdSe and CdSe/ZnS where the ZnS shell was still sufficiently thin enough (2.5 monolayers) to permit competitive hot hole extraction (with transfer times of 5 ps and 20 ps respectively). In that case the anticipated application was in QD doped solar cell devices where the type I shell would improve photostability. Type II and quasi type II where the electron can range over both core and shell, whilst the hole remains localized in the core or the converse case where the electron is confined to the core whilst the hole can move throughout the structure have proved to be of far greater interest for CM. Pandey and Guyot-Sionnest first used type II structures with further additional layers to allow holes to be localized in and even trapped at thiol ligand sites on a thin outer CdSe shell (CdSe/ZnS/ZnSe/CdSe/thiol). Confinement of the electron in the core, without access to a hole to transfer excess energy to, resulted in cooling times being extended from under 6 ps to over 1 ns. Similar frustration of the cooling process has been seen in PbSe/CdSe core/shells with thick CdSe shells [3,105,106]. In this case it is the holes that are strongly confined in the small core, with a concomitant increase in the core valence level spacings. In addition, the overlap between the lower energy core and higher energy shell valence levels is reduced further slowing hole relaxation. The presence of the CdSe shell brings a benefit that helps to offset the very similar hole and electron effective masses that leads to the high CM threshold in lead chalcogenide QDs. Absorption at higher energies is more dominated by the CdSe shell leading to a non-equal partition of excess energy between the two photogenerated carriers. Excitation near the shell band edge therefore bestows far more excess energy on the holes which is said to lead to more efficient CM, i.e., a fourfold increase in CM efficiency (relative to equivalent sized core only PbSe QDs) and a reduction in threshold energy to close to 2 E_g_ [106]. In these core/shells, weak visible emission at close to twice the IR band edge emission is also visible, showing evidence of the preservation of hot holes long enough for detectable hot carrier recombination to occur.

A similar hole in core confinement scheme was used by Gachet et al. [107] using type II structures based on CdTe/CdSe core/shells (with additional shell layers of CdS and ZnS). At low excitation energies, below either the CdTe or CdSe band edges, weak CM was tentatively identified which was interpreted as arising from CM via a spatially indirect process across the core-shell interface, at least in part.

InP/CdS quasi type II core/shells have also been investigated by several groups [20,103]. Smith et al. [103] grew 3.9 nm diameter InP cores coated with 0.7 nm thick CdS shells with both core and shell in cubic phases. CM QYs (122% at 3 E_g_) were similar to that for type I InP/ZnS/ZnO QDs [108], and biexciton lifetimes were measured as 50 ps, broadly similar to those determined by Dennis et al. [20] for the thinner shell samples in a series with thicknesses ranging from 1 monolayer up to 11.4 monolayers of CdS. For their thicker shell materials, the biexciton lifetimes (determined from time resolved PL measurements) increased to 7.2 ns ± 1.1 ns showing a dramatic reduction in Auger recombination and a similar abatement of QD emission blinking. However, the authors noted that the ratio of the single exciton (702 ns) to biexciton lifetimes is still far higher than the theoretical ideal case of 1:2 to 1:4, indicating that whilst Auger recombination is drastically suppressed, it is still not completely absent as a non-radiative recombination channel. Dennis et al. mentioned that their core/shells have InP in its cubic phase but that the overgrown CdS shells adopt the room temperature stable hexagonal phase, with less than 1% lattice mismatch at the core-shell boundary.

### 5.4. Surface Effects

It has long been recognized that QD surfaces can substantially modify carrier dynamics. Imperfect surfaces may provide lattice defects that act as traps for either electrons or holes, or generate additional phonon modes that may couple to the carriers [102]. Even a perfectly formed surface must act as the interface between the interior lattice and surface ligands, bringing the possibility of coupling to the molecular vibrations of the latter, often this is very evident in low bandgap IR QDs [109,110,111]. In regard to CM, the surface can influence the multiplication process in two ways: the carrier cooling processes that compete with CM for hot carriers can be modified, as shown by Pandey and Guyot-Sionnest [102] where photogenerated holes were intentionally localized on the surface ligands of CdSe/ZnS/ZnSe/CdSe heterostructures, leaving electrons to cool much more slowly than normal without being able to transfer energy to the holes via Auger cooling channels. The authors also noted major differences in TA signal decay times between samples terminated with alkane thiols or amines and ligands such as phosphonic or carboxylic acids. The former, having weaker and less extensive mid-IR spectra provide less scope for surface coupling and energy loss to molecular vibrations than the latter. This exciton-ligand interaction then furnishes a second cooling mechanism, distinct from regular Fröhlich type carrier-phonon relaxation. The other way in which CM can potentially be affected by the QD surface is by modification of the CM rate itself, for example by the opening of additional impact ionization channels via defect states appearing in the gap [112].

The influence of the oxidation of PbS QDs to form a surface layer of PbSO*_x_* [113,114] and its impact on CM yields, thresholds and carrier extraction efficiencies was investigated by Hardman et al. [115]. They found evidence of a reduction in CM efficiencies and an increase in threshold energies, along with a reduction in the carrier injection efficiency that correlated with the degree of surface oxidation, probed by XPS characterization of the surface species.

We have of already mentioned above that photocharging can masquerade as MEG and Auger recombination signals [116], and in many cases the degree of photocharging and its kinetics can be linked to differences in surface states and surface treatments between different samples [19,86]. Midgett et al. [81] inferred a size dependent hot carrier cooling rate in addition to a size dependent CM rate in their studies on PbS, PbSe and PbS*_x_*Se_1−*x*_ alloy QDs which they suggested may be tied to variations in stoichiometry with size, imperfect surfaces or changes in ligand coupling modifying the competing cooling channels. Spoor et al. [54] also note the potential impact of the choice of ligands and exciton-molecular vibration coupling on the cooling rate in their detailed study of the hole and electron cooling spectrum in PbSe QDs (see Figure 5). Near the band edge in particular, a cooling mechanism via coupling of both electrons and holes to surface ligand vibrations or to surface phonon modes of the QD itself is a necessary conclusion. As such, the choice of ligand (due to its IR overtone and combination band spectrum) will have a significant effect on the cooling rate and therefore the competition between cooling and CM.

CM and carrier dynamics have also been studied in Ag_2_S, CuInS_2_ and CuInS_2_/ZnS core/shell QDs by Sun et al. [117,118]. Here the radiative recombination mechanism is slightly different from that in the II–VI, III–V and IV–VI QDs in that it is associated with internal defect states e.g., Cu vacancies in CuInS_2_. Carrier dynamics are very sensitive to the surface where charges (holes in particular) may become strongly localized (or trapped) and the surrounding medium polarity can also exert a large effect. In CM measurements on Ag_2_S QDs, Sun et al. [117] reported that two different types of Auger processes were present, one involving relaxation of tightly bound excitons and the other weakly bound excitons. The proportions of each type of recombination was found to be sensitive to the polarity of the solvent the QDs were dispersed in: lower polarity favoured the weakly bound excitons, where the hole was determined to be localised near the QD surface, whilst QDs dispersed in higher polarity solvents had a more equal proportion of both types of exciton. CM threshold was 2.28 E_g_, with an efficiency of 173% measured at 3.2 E_g_. Stolle et al. [50] reported a CM threshold of 2.4 E_g_ and an efficiency of 36% per unit of E_g_ above the CM onset in CuInSe_2_ QDs. Sub-CM threshold excited carrier cooling rates were in the 1 eV/ps range and exhibited a QD volume dependent scaling.

### 5.5. Doping/Photodoping Effects

QDs may be doped in order to selectively alter their carrier mobilities or to modify their electronic and optical properties. They may be manipulated by impurity doping [119,120] just as in bulk semiconductors to be n- or p-type, or they may be electrochemically doped by a combination of surface treatments [121] and charge injection via electrodes [122] or contact with electrolytes in electrochemical cells [123,124] Another strategy is so-called photodoping, where partial population of a lower lying excited state is effected by optical excitation in advance of an event such as a pump and probe pulse during TA measurements, etc. In all cases, the normally vacant excited states of the QD are partially filled. This can be useful in solid state QD solar cells where p and n type layers (with one or both being doped QDs) can lead to the formation of heterojunctions with a built-in field to drive charge separation and transport towards the electrodes. Doped QDs can be used to open up normally inaccessible intraband transitions [125] with energy level spacings that can be used to extend the IR range of QD photodetectors [126] for example. In QD lasers, partial filling of the degenerate upper lasing level can reduce the excitation threshold for laser action to occur [123]. Given the interest in doped QDs for solar cells in particular, it is of interest to know what effect doping may have upon CM performance, i.e., does one compromise the other? Two possibilities might arise: additional intraband transitions from the populated band edge states could open up further channels for CM; or, where the doping populates the band edge state either fully or one carrier short of fully, transitions to biexciton states in that level can become blocked according to the Pauli exclusion principle (Pauli blocking) [46,123]. In the CM context, multiplication would then be blocked until much higher excess energies where higher levels could be filled by carrier fission. Pijpers et al. [30] attempted to show evidence of just this blocking mechanism in relation to CM in InAs/CdSe/ZnSe core/shell QDs by photodoping the 1S_e_ electron transitions with a leading pulse a few ns before measurements of sufficient fluence to ensure that by the time the measurement was made, the 1S state of the ensemble was almost completely singly populated. With a degeneracy of two for the 1S electron states, CM can lead to a triexciton state (the original leading pulse exciton plus a further two excitons from CM, one into the 1S and one into the 1P states). This would alter (increase) the CM threshold, and the CM biexciton creation energy. Initially the authors reported a reduction in CM efficiency from 1.6 to 1.3, however, they were subsequently unable to repeat the CM experiments with fresh InAs/CdSe/ZnSe QD batches that were nominally identical to the previous materials. The CM part of the earlier reported work was consequently withdrawn [30] leaving the photodoping demonstration open to question.

### 5.6. Alloy Composition Effects

The engineering of bulk semiconductors for the enhancement of CM is limited to the formation of superlattice structures [2] or manipulation of the band structure around the bandgap by the formation of alloys. The latter has been successfully applied to Ga_1−*x*_Al*_x_*Sb, InGaAs and Hg_1−*x*_Cd*_x_*Te in particular in relation to the production of APDs [4,6]. In the zincblende Ga_1−*x*_Al*_x_*Sb and Hg_1−*x*_Cd*_x_*Te cases, the composition is adjusted to bring the split-off valence band and the band gap energy differences (Δ and E_g_ respectively) into resonance to improve the impact ionization efficiency. This resonant condition is nearly true for bulk InAs without resorting to the use of alloys [4]. For direct semiconductors satisfying this condition the impact ionization process is vertical and with no momentum transfer the threshold can approach the 2 E_g_ energy conservation limit.

**Figure 14:**
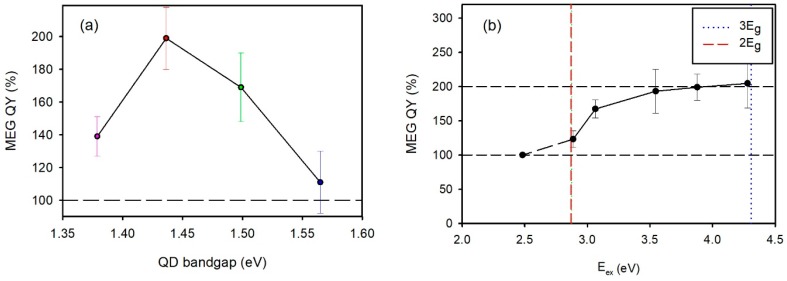
(**a**) MEG QY vs. Cd*_x_*Hg_1−*x*_Te alloy QD bandgap energy (E_g_) for a range of compositions, at excitation energy 4.28 eV, measured using the TG method. The peak QY obtained was 199 ± 19%; (**b**) MEG QY threshold curve for the sample corresponding to the red symbol in figure (**a**). The data for (**b**) supplied courtesy of Prof. Q. Shen and colleagues [131].

In QDs there is an additional degree of flexibility in that as well as being able to tune the composition, the size can also be easily adjusted. This then means that the resonance condition could be matched for a wider range of bandgap energies, though the tuning of the two energy level differences, Δ and E_g_, is not completely independent. In QDs, the notion of a split-off valence band is superseded by more discrete valence band levels, but the spirit remains the same. The energy gap “Δ” can be determined by spectroscopic ellipsometry if good optical quality thin films of QDs can be prepared [127,128]. In a previous study of CM in Hg_1−*x*_Cd*_x_*Te QDs prepared by ion exchange [129,130], we estimated a size/composition point where a strong resonance could be expected and then bracketed that point by preparing a series of same-sized but different composition alloys starting from a single batch of CdTe QDs. A composition sweet spot was observed where the CM QY (determined by the TG method) reached almost 200% for excitation at 2.9 E_g_ (Figure 14a). At this point the composition was *x* = 0.52, and the m_e_/m_h_ ratio would be around 0.14 which would lead to a prediction of a low *E_th_*, close to the energy conservation limit. Subsequent threshold excitation energy dependence measurements revealed a threshold of close to 2 E_g_, with evidence of saturation between 2.5 E_g_ and 3 E_g_ (Figure 14b).

Midgett et al. [81] also observed CM in alloy QDs of PbS*_x_*Se_1−*x*_ though in the lead chalcogenides the lattice is hexagonal and so the degeneracy of the band edge states is higher than cubic Hg_1−*x*_Cd_*x*_Te. This should allow far higher exciton multiplicities to be reached well above *E_th_* though no reports to date have shown evidence of a staircase-like rise in CM QY vs. excitation energy. In all cases (not just alloys) a linear data fit prevails to at least 3 E_g_ if not higher.

## 6. Conclusions and Outlook

Since the first observations of CM in QDs, the understanding of the underlying carrier fission process and the competing relaxation channels has come a long way, though there is undoubtedly still more to learn about the latter. The possibility of several simultaneous carrier cooling mechanisms has complicated the unravelling of the carrier dynamics and even now there remains some conjecture even for simple QD structures, particularly when it comes to the role of surface polarons and ligand molecular vibrations in dissipating the initial hot carriers’ excess energies. Refinement of measurement and analysis techniques has removed some of the wide variations in the early reported CM data, allowing a clearer picture to emerge. In particular, the comparison of symmetric (in terms of the carrier’s effective masses, meaning m_e_~m_h_) QDs such as the lead chalcogenides with asymmetric QDs (m_e_ << m_h_) such as CdSe and InAs allowed the role of Auger cooling (electron to hole energy transfer) in the latter to be fully appreciated. The observation of an engineered phonon bottleneck in CdSe/ZnSe core/shell QDs further supported the development of this thinking. Detailed carrier cooling studies in the lead chalcogenides have highlighted the influence of surface phonons/ligand vibrations in phonon mediated cooling processes, which seem to outpace the classic bulk LO and acoustic phonon mechanisms, and which could explain the cooling rates seen experimentally [54]. The further development of CM has and will continue to focus not so much on the multiplication mechanism itself (though several such mechanisms are postulated) as on minimization of the net competing cooling rate. To date, the effects of composition, size, the use of nanostructures and dimensionality have all been explored with a view to slowing each of the cooling processes. Once multiplication has been given the chance to occur, the resulting multiexciton must then resist non-radiative recombination, particularly via Auger recombination, this being effectively the inverse of the CM process. Here again, the use of heterostructures, or higher dimensionalities such as in nanorods and nanoplatelets have been shown to enhance biexciton and multiexciton survival.

For applications such as solar cells and photodetectors which rely on extraction of the hot, or in the case of CM the enhanced numbers of, carriers there are certain time windows for that extraction. If hot carriers are to be extracted [17], the charges must be removed before cooling, so that CM and any kind of Auger recombination cannot occur. CM is a fast, tens to hundreds of fs, process whilst cooling overall can often range from sub- to a few ps in core-only QDs. Thus, for hot carrier extraction it is probably better to select materials which do not themselves show efficient CM. For multiexciton extraction following CM, the removal of charges must come after the hot exciton fission but before multiexciton recombination, and in some of the materials seen to date this can be extended into the few ns regime, which is a less difficult prospect than for hot carrier extraction. Whilst we have not covered the extraction or transfer of charge to adjacent acceptors in this review, it is a very active field of research and there are many reviews [132], and recent research papers [133] on this subject. Of course, the efficient transport of the carriers through the QD or QD/host matrix film once they have been carefully harvested is an equally, if not arguably a more, important subject which also continues to be investigated [134,135,136,137].

To make an appreciable impact on QD solar cell power conversion efficiencies, overall CM still needs to improve to be much closer to the ideal limit to be useful [14]. QD solar cells without CM still have power conversion efficiencies around the 10–12% mark [138,139], far below the Shockley-Queisser limit [12], so clearly as a field we must continue to address the basics such as carrier extraction and transport. Some examples of CM enhancement of device photocurrent exist [15], but the improvements are still relatively marginal and require careful measurement methods to unambiguously allow them to be attributed to CM. Calculations have shown [13] that simply being able to observe CM at some level in a QD solution does not necessarily translate into a quick fix for QD solar cell efficiencies. A lot more development to bring CM up to the ideal in terms of a near energy conservation limited threshold and much higher CM efficiencies (i.e., following the staircase-like characteristic) is still required.

The newly emerging field of lead halide perovskite nanocrystals and nanoplatelets [140] offers some potentially interesting prospects for future exploration of hot carrier extraction and CM. The occurrence of traps within the bandgap is known to be uncommon, or at least limited to very shallow trap states so such nanoparticles are termed to be defect tolerant [141,142,143]. With traps occurring within rather than between the conduction and valence bands, the exciton QYs can be very high (>90%) even without the use of core-shell passivation. This suggests that photocharging may be somewhat reduced in these materials, as well. Biexciton lifetimes of 90 ps in perovskite nanocrystals have been observed by Hu et al. [144] Interestingly, a hot phonon bottleneck has already been seen in thin films of bulk perovskites [145], and has been observed to be far more effective at slowing carrier cooling than in some regular epitaxial semiconductors such as GaAs [146]. The origin of the slow, hot hole cooling has been explained theoretically as originating from the relatively sparse density of phonon states in the valence band [147]. Li et al. [148] have already demonstrated that this slow cooling translates into colloidal perovskite nanocrystals, with cooling rates up to two orders of magnitude slower than for films—cooling times of up to 20 ps were seen at low fluence whilst at higher fluences an Auger *heating* process contributed to extending this to 27 ps. This allowed the authors to demonstrate efficient (83%) hot carrier extraction in under 1 ps using an organic electron acceptor molecule. Already, several groups have shown that core-shell structures, although less straightforward than in II–VI QDs, etc., and so far with a more restricted choice of band offsets, can nonetheless still be grown [149,150]. The growth of 2D perovskite nanoplatelets has also been demonstrated [140,151], so there is already a toolkit of structural options that can be explored to further manipulate carrier cooling rates to favour CM. However, for CM tailored to solar applications the perovskite bandgap would need to be best positioned just into the near IR (e.g., 1.2 eV–1.4 eV). This is still a slight problem for many perovskite NCs, where even red emitting materials tend to be metastable. Protesescu et al. [152] have recently broken through this so called ‘red wall’ by using formamidinium-cesium lead iodide to form more stable 780 nm emitting materials, offering a further step towards solar cell applications for perovskite nanocrystals. To date there have been a few attempts at the demonstration of CM in perovskite nanocrystals, however, despite the large exciton binding energies which should favour high CM rates [153], multiplication has not been observed up to 2.65 E_g_ whilst the biexciton and trion Auger recombination rates have been faster than in their II–VI counterparts [154]. However, there is much scope for future improvements with nanostructure engineering using the lessons already learned from II–VI, IV–VI and III–V nanoparticles.
